# Mining Productive-Associated Periodic-Frequent Patterns in Body Sensor Data for Smart Home Care

**DOI:** 10.3390/s17050952

**Published:** 2017-04-26

**Authors:** Walaa N. Ismail, Mohammad Mehedi Hassan

**Affiliations:** Information Systems Department, College of Computer and Information Sciences, King Saud University, Riyadh 11543, Saudi Arabia; 435204202@student.ksu.edu.sa

**Keywords:** body sensor network, smart home, knowledge discovery in BSN data, frequent patterns, periodic patterns, productive pattern

## Abstract

The understanding of various health-oriented vital sign data generated from body sensor networks (BSNs) and discovery of the associations between the generated parameters is an important task that may assist and promote important decision making in healthcare. For example, in a smart home scenario where occupants’ health status is continuously monitored remotely, it is essential to provide the required assistance when an unusual or critical situation is detected in their vital sign data. In this paper, we present an efficient approach for mining the periodic patterns obtained from BSN data. In addition, we employ a correlation test on the generated patterns and introduce productive-associated periodic-frequent patterns as the set of correlated periodic-frequent items. The combination of these measures has the advantage of empowering healthcare providers and patients to raise the quality of diagnosis as well as improve treatment and smart care, especially for elderly people in smart homes. We develop an efficient algorithm named PPFP-growth (Productive Periodic-Frequent Pattern-growth) to discover all productive-associated periodic frequent patterns using these measures. PPFP-growth is efficient and the productiveness measure removes uncorrelated periodic items. An experimental evaluation on synthetic and real datasets shows the efficiency of the proposed PPFP-growth algorithm, which can filter a huge number of periodic patterns to reveal only the correlated ones.

## 1. Introduction

In recent years, the use of body sensor networks (BSNs) to remotely monitor and collect the vital sign data of patients to extract knowledge on their health condition has become an effective solution in a smart home environment to enable the growing number of elderly as well as physically impaired people to stay alone in their homes with full care and support [1,2,3,4]. With several ambient sensors, wearable sensors, and biomedical devices implemented throughout the home as well as wearable health trackers, the resident’s health conditions are continuously monitored and any urgent assistance is triggered when an abnormal situation is detected. Depending on the situation, healthcare caregivers can receive warnings or help alerts from healthcare service providers. A smart home goal is to help the elderly and people with disabilities feel comfortable and practice their daily activities on their own, while monitoring their safety and well-being. This improves the elderly’s feelings and reduces the cost imposed on society and the healthcare system.

With the improvement in health consciousness and advances in BSNs that are used to collect the vital signs of a patient in their smart home environment, there is a need for new approaches and systems to address healthcare monitoring and decision making [5,6,7,8,9,10]. BSNs can be used to extract knowledge about the health condition of the patient and these have enabled the development of many real-time activity recognition approaches [11,12,13,14]. Recently, most research has focused on human activity recognition through wearable devices and BSN-generated data to monitor and follow up patient health. The analysis of BSN-generated data can enable the early detection of unusual activities or abnormal health conditions by monitoring the daily living activities of users (e.g., elderly, cognitively impaired people, patients). Moreover, the behavior profile parameters sensed by the body sensors provide knowledge for doctors to treat a particular patient. For example, identifying periodic changes in the body temperature or heart rate of a patient can be useful information. Thus, the discovery of the shape of a pattern's occurrences (i.e., periodic or partial periodic) and the relationship between the physiological information obtained from the BSN can help predict or provide care to the user.

However, using pattern matching [15,16] or human daily activity recognition [11,12,13,14] algorithms to find such important and interesting knowledge from BSN readings may be unsuitable because of the high data rate and variety of data streams obtained from BSNs. Recently, data mining techniques that aim to discover new knowledge from the obtained data have also been utilized to analyze knowledge from the BSN data [17,18,19,20]. A software architecture was developed in [19] to monitor routine behavior based on a patient’s daily activity. This obtains frequent patterns in order to identify the structure of a human’s daily activity using a frequent pattern-mining [21] technique. An automatic data mining method using physical activity measurements was proposed by Candás et al. [22] to detect abnormal human behavior. Machado et al. [23] designed a human activity recognition framework using on-body accelerometer sensors. Nevertheless, all these pattern-mining approaches are limited in terms of detecting periodic changes in human behavior or identifying a subject’s activity.

Furthermore, periodic-frequent or regular-frequent pattern mining, which aims to discover those frequent patterns that occur at regular intervals in a temporally ordered transactional database, was studied by Tanbeer et al. [24,25,26] with the aim of identifying frequent periodic patterns since the shapes of a pattern's occurrence in databases cannot be determined by the interesting measures (such as support and closure) used in frequent pattern-mining approaches. Additionally, Rashid [27] proposed a different measure (regular-frequent pattern mining), measured as the variance among frequent pattern periods, in order to detect periodic patterns in transaction-like databases. On the other hand, Tanbeer et al. [24,25] introduced an efficient approach to detect and identify regular behavior patterns that exhibit complete cyclic repetitions from BSN data. It uses a periodic pattern-mining algorithm to analyze patient data in order to follow up the health conditions of patients. As the real world is generally imperfect, some interesting patterns that occur frequently with partial cyclic repetitions in humans’ daily routines may exist and those patterns may have a significant effect on human health and could help caregivers take serious decisions regarding a patient’s health [28]. Unfortunately, those types of patterns cannot be identified using the existing periodic-frequent pattern-mining algorithms because the approaches in [24,25,26] try to discover those patterns that are frequent and have complete cyclic repetitions in the entire database. Most of these algorithms use a maximum periodicity threshold to discover periodic patterns, which measures pattern periodicity based on the largest amount of time difference or number of timeslots between two occurrences of a pattern. Typically, a pattern with single periodicity greater than the user-defined maximum periodicity threshold will be discarded and it will be considered as non-periodic. This approach is not flexible, as some interesting patterns can be discarded based on only one of its periods. In this paper, we propose a solution to this problem by discovering periodic patterns using novel measures: the interested-recurrence period, minimal-itemset occurrence, and average interested-period to assess the periodicity of patterns. 

In addition, data analysis in the case of BSN is no easy task. Consider, for example, the case of caregivers or doctors being interested in identifying the set of vital health parameters with similar occurrence periods that occur nearly in the same time periods. On the other hand, some of a patient’s periodic (regular) frequent patterns are periodic due to random sensor readings without inherent association. Using such periodic-frequent patterns without analyzing item associations could be detrimental for caregivers in decision making about a patient’s health. To overcome this challenge, we employ a productivity test to identify the set of productive-associated periodic-frequent patterns.

**Example** **1.** *During human profile analysis, the caregiver observed from the set of readings that the patient’s heart rate and blood pressure status were very high and less frequently-occurring than the high readings of blood pressure and body temperature. Furthermore, the duration between two consecutive high readings can be generally longer than for two consecutive high readings of blood pressure and body temperature. Identifying the above types of inherent regularities in a patient’s health-related readings can be significantly helpful for caregivers in following up a patient’s health condition and enabling real-time health-related data analysis.*

In [28], the authors introduced a new class of patterns known as chronic-frequent patterns by investigating the partial periodic behavior of frequent patterns in a transactional database. A pattern is said to be chronic frequent if it has a sufficient number of cyclic repetitions in the entire database. The method uses a pattern-growth mining approach with two database scans, which cannot be applied in the case of BSN-generated data because of the high volume of data that need to be read once. In addition, the number of generated patterns is huge, partially because of random occurrences without item relationships. The work of [29] introduced a new type of periodic pattern named productive patterns, which have the ability to find full period patterns (i.e., patterns that occur at regular intervals), and conducted a productivity test to ensure the association between the generated patterns. Nevertheless, the approach uses a generate-and-test method based on the a priori algorithm, which results in a huge search space and thus cannot be applied in the case of stream data [21]. In our current work, we employ the productivity test using an efficient fp-growth-inspired approach to mine the set of productive patterns.

In this paper, we plan to develop a smart home solution that maintains the occupant lifestyles of care users, especially the elderly and physically impaired people. We aim to achieve this goal using sensor-based data collection systems (e.g., BSNs) relevant to a “smart home” for efficient healthcare decision making. With this motivation, we propose an approach to identify the periodic interestingness of health-related vital signs in a single run using an efficient pattern mining algorithm. Considering the productivity of all periodic vital signs and with the elimination of randomly generated patterns, the overall system helps reduce false alarms in monitoring stations. In this paper, we introduce a new tree structure, called the Productive Periodic-Frequent Pattern Tree (PPFP-tree), to capture both the frequency and the periodic behavior of the patterns. A pattern-growth algorithm, called Productive Periodic-Frequent Pattern-growth (PPFP-growth), is then proposed to discover the patterns from the PPFP-tree. Our contributions are as follows:

1. We focus on mining the different parameter readings obtained from body sensors that occur either fully or partially in the smart home in order to follow up a patient’s health conditions using a novel tree-based data structure, called the PPFP-tree, with a single database scan.
2. We further employ two pruning techniques. The first technique is based on the concepts of partial periodic patterns and is used to discover periodic-frequent patterns containing either cyclic or acyclic pattern repetition. The second one is a productiveness measure used to ensure that periodic-frequent patterns without item associations or more generally obtained due to random occurrences are eliminated.
3. Once the PPFP-tree is constructed, we use an efficient pattern-growth-based mining technique to mine the patient readings (PPFP-growth) algorithm using our pruning techniques.
4. A performance study is conducted to compare the performance of PPFP-growth with existing periodic mining algorithms, and we show that PPFP-growth is more runtime-efficient than existing algorithms.

The rest of the paper is organized as follows. Section 2 presents an example scenario for applying the proposed approach in BSNs. Section 3 presents related work. Section 4 introduces our model of mining productive-associated patterns. Section 5 describes the working of the PPFP-growth algorithm. The experimental evaluation of PPFP-growth and some recent related work is presented in Section 6. Finally, Section 7 concludes the paper and provides directions for future work.

## 2. Productive-Associated Periodic Pattern Mining in Healthcare

Consider an assisted living system where a patient lives alone and let a set of body sensors be attached to a patient’s body to obtain health-related data continuously (i.e., every minute). Each sensor will collect a particular type of vital sign as shown in Table 1.

Thus, the sensor readings obtained by all sensors (e.g., HR, RR, SO_2_, DBP, and BT) can be shown as a combination of the different vital signs from all sensors (e.g., a vital sign reading list). Depending on the ranges and/or types of vital parameters, the values sensed by each sensor can be subdivided into several categories based on a predefined range, as shown in Table 1. For example, if the readings from HR and BT are HR-High and BT-High at time Tn, respectively, the vital sign reading list for Tn would be as follows: Tn: (HR-High, BT-High). Thus, the readings continuously generated by the sensors for the patients can be represented as:

T1: SO_2_-Low, RR-Low;
T2: DBP-Normal, HR-High, BT-Very High;
T3: HR-High, BT-High;
T4: SO_2_-Low, RR-Low;
T5: DBP-Very High, HR-Low;
T6: HR-High, BT-Low;
T7: DBP-low, HR-Very High, BT-Low.
T8: HR-High, BT-High;
T9: SO_2_-Low, RR-Low.

Once the temporal readings are recorded in the form of the above lists with timestamp information, we can apply our PPFP-tree to obtain all periodic patterns in patient data, as patients who are actually ill are likely to have several abnormal periodic vital signs. For example, it can be observed from the above set of readings that the pattern < SO_2_-Low, RR-Low > occurs three times in the patient reading vital sign at (T1, T4, T9), which may indicate that the blood oxygen saturation and respiratory rate decreases at least once during the daytime. Once we find this pattern, we apply our productivity test to see to what extent SO_2_ and RR have a productive association. Once we discover such knowledge, the caregiver will easily detect that the patient will experience hypoventilation [30] at any time. Moreover, the monitoring caregiver can identify additional patterns of abnormalities by monitoring the productive-associated periodic vitals lists. For example, a serious emergency known as sepsis can occur when the patient has low blood pressure, a very high heart rate, and a decrease in body temperature. Therefore, caregivers need to be aware of these abnormalities to avoid any potentially emergent situation associated with periodic changes in multiple vital signs.

## 3. Related Work 

The flexibility and widespread use of wearable sensors has promoted the process of modeling human behavior from sensor-generated data for the purpose of following human health and detecting any unusual or emergent behavior through human recognition systems that can monitor occupants’ behavior in smart environments. The research community has presented several frameworks for prototyping general smart environments or particular smart homes. As an example, Evangelatos et al. [31] proposed the *Syndesi* framework. *Syndesi* creates a personalized smart environment to realize human actions based on human behavior profiles. The framework provides the necessary services to home occupants to quickly control and react easily with their environment (e.g., electrical devices based on human preferences and needs). Rom´an et al. [32] proposed *Gaia*, a distributed middleware supporting ubiquitous computing environments. *Gaia* allows developers to monitor a combination of individual services as a whole by introducing programmable active spaces. In the context of the *CASAS* project, Rashidi and Cook et al. [33,34] presented automatic daily life activity pattern discovery and monitoring for assisted living. This overcomes the limitation of supervised activity-recognizing approaches by discovering human activity from collected sensor data. Additionally, the system can detect variations in human monitoring services and is also able to handle real-time multi-sensor data efficiently. Recently, many cloud-assisted frameworks for the development of smart homes have been proposed by the research community [35,36,37,38]. The work in [39] shows *CASE*, a framework that recognizes human activities by discovering the frequent episodes from occupant-related data based on the a priori algorithm [40]. The work in [41] shows a probabilistic-based approach to forecast smart home occupants’ behavior and wellness by monitoring the daily usage of home appliances. For example, elderly people’s behavior follows some regularity in their activity execution such as eating at 7 a.m. and sleeping at 9 p.m. When daily activities are performed regularly, it means that the smart home inhabitant is in a state of wellness. In this work, ambient-assisted predictive techniques are used for the extraction of patterns related to sensor activation time. These projects monitor human’s regular living activities, using heterogeneous obtrusive camera sensors for accurate identifications of routine activity’s contextual aspects. The main obstacles in deploying such project technologies are installation complexity, high costs and privacy obtrusiveness related issues [19,33]. Recently, many works in the areas of human behavior analysis [14,15,16,17] and human activity recognition have been conducted; unfortunately, however, most of those methods require supervised techniques for handling and labeling sensor-generated data. Additionally, real-time human activity recognition using data-mining techniques has become popular for detecting human behavior from BSN data [17,18,19,20]. Candás et al. [22] proposed an unsupervised data mining method to detect unusual (abnormal) human behavior using physical activity. This detects anomalies in behavior automatically by assessing the physical activity level of human and compares it with existing historical data.

Despite the accuracy of the proposed approaches in activity recognition, a significant proportion of smart home services provided by a smart home comprises monitoring residents’ activities to find only frequent patterns in their activities of daily living for health purposes. Hence, a significant amount of research is still required to develop algorithms that can help with an accurate diagnosis [42,43,44]. Moreover, frequent patterns are huge in space if we try to find them in BSN-generated data and are limited in terms of detecting changes in human behavior that occur regularly or periodic in everyday life.

Nevertheless, mining periodic-frequent patterns is no easy task as it is faced with several challenges. For instance, the periodicity measure in [27], which is susceptible to noise in the database, might often report the noised maximal period of a pattern as its regular period. Additionally, as we mentioned earlier, the methods in [25,26] often generate regular (periodic) frequent patterns that occur in the whole database with totally distinct periods.

Recently, Tanbeer et al. [26] developed a regular pattern tree to precisely mine regular patterns from transactional databases. This approach requires two database scans and uses the maximum occurrence interval of a pattern in a database to measure a pattern’s periodicity. Thus, many researchers are extending Tanbeer’s work to mine top−k [45,46,47] periodic patterns, but their approaches remain limited to k items. The work presented in [24,25] proposed an efficient and scalable regular mining algorithm with one database scan. The algorithm can be conducted in either single or multiple distributed BSN data for the purpose of following up the health conditions of users. A major drawback of those approaches is using a maximum periodicity threshold as a measure for finding periodic patterns, which results in discarding the itemset automatically if it has a single period of length value greater than the *MaxPr* threshold. Thus, this measure is too strict and could lead to losing a very important pattern that appears irregularly or partially.

In [48], the authors introduced a periodic-ratio measure to report the partial periodic pattern that occurs frequently in a transaction-like database. Unfortunately, the periodic-ratio measure does not satisfy the downward closure property, and as a result the approach is computationally extensive. In [28], the authors reported a kind of periodic pattern called a chronic-frequent pattern, namely frequent patterns that have either complete cyclic repetition in the database or partial cyclic repetition. The model builds a chronic-frequent tree with two database scans. In [29], a productive periodic pattern is generated; productive patterns are exhibited frequently, and this regularity is not due to the random occurrences of uncorrelated items. Further, the framework in [29] limits the pattern’s periodicity to a given threshold and within the same range of a period’s values given by the user. The model uses the basic a priori-like approach to generate the periodic pattern.

Again, some of the mentioned work considers both partial and full cyclic pattern mining with two database scans. However, none of the above algorithms can be applicable to efficiently mining the large volumes of data coming from BSN data, which are close to transactional databases, to detect the correlated periodic patterns that occur in partial or full cyclic databases. Such correlated patterns could help detect some new knowledge, especially for human care or disease detection. Therefore, there is a need to develop an efficient mining technique to address the problem of productive pattern mining from body sensor data. 

Table 2 compares our approach and related approaches with respect to three issues: (i) the ability to discover full or partial patterns, (ii) the ability to discover patterns using one database scan, and (iii) the discovery of correlated periodic-frequent patterns. It can be observed that our approach tries to address all of the issues, while related work addresses only some of them. 

## 4. Proposed Model

**Figure 1:**
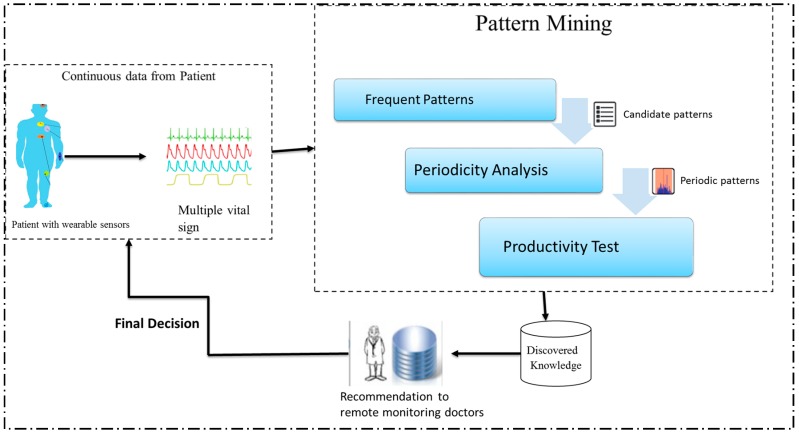
The workflow of productive-associated periodic-frequent pattern mining.

Our approach to remote monitoring systems is presented in Figure 1, where an elderly person is equipped with different body sensors (e.g., ECG sensor, BP sensor). These sensors collect different physiological data on the patient in a continuous manner. After processing vital data to a predefined data format, which is understandable to the proposed data-mining algorithm, Productive-Periodic-frequent pattern (PPFP-tree) data structures will be used to capture the processed epochs from the input stream data in a fixed order satisfying the user-specified threshold parameters for periodicity and frequency. Moreover, the PPFP-tree will efficiently preserve the temporal information (timeslot id) of each epoch. Once the underlying sensors’ data are captured in the PPFP-tree, an efficient data-mining algorithm (PPFP-growth) will be applied in order to completely mine the complete set of periodic patterns. The mining technique will automatically discover the interesting itemsets along with its temporal information. The user-specified frequency and periodicity threshold will be pushed to the mining process to eliminate unnecessary patterns. Furthermore, the productivity test will be used to reveal only the correlated periodic-frequent patterns. The periodicity and productivity of each pattern will be identified based on the extracted temporal information for the pattern. The results of the mining process are used to recommend a patient’s doctors for subsequent decisions related to the patient’s health condition. 

The basic notations and definitions of productive-associated periodic pattern mining in a body sensor database are as follows:

Let a set of body sensors represented as T = {$BS_{1}$,$\;BS_{2}$, …, $BS_{n}$} be in a particular BSN at smart home SM. The pattern of a sensor’s X = {$BS_{j}$, …, $BS_{k}$} ⊆ T, where j ≤ k, is of length-k, satisfying some conditions of measures such as frequency; for instance, X = {$BS_{1}$,$\;BS_{2}$, $BS_{3}$} is a length-3 pattern. 

A body sensor database, BSD, over T is defined to be a nonempty set of an epoch’s BSD = {$t_{1}$, …, $t_{m}$}, where each epoch in the BSD is identified by m (called TID), where TID represents the occurrence of the timeslot-id of the sensor. The cover of pattern X in SDB,$\;cov_{BSD}\left(X\right)$, is the set of epochs’ TIDs that contain X. That is,

(1) $$cov_{BSD}\left(X\right)=\left\{m\;:t_{m\;}\in BSD\wedge \;X\subseteq t_{m}\right\}$$

The support of a pattern X in $\mathrm{SDB}\;Sup_{SDB}\;\left(X\right)$, is defined as:

(2) $$Sup_{SDB}\;\left(X\right)=\;\frac{\left|cov_{BSD}\left(X\right)\right|}{\left|SDB\right|}$$

where |$cov_{BSD}\left(X\right)$| is called the support count of X in SDB and | SDB| represents the size of SDB in the total number of epochs. Pattern X in SDB is said to be frequent if $Sup_{SDB}\;\left(X\right)$ is larger than or equal to minSup(ε), a user-specified minimum support threshold value.

**Example** **2.** *Consider the sensor database shown in Table 3* *. It contains seven epochs. The set of items, I = {* $BS_{1},BS_{2},BS_{3},BS_{4},BS_{5}$ *}, and the set of body sensor readings* $\prime BS_{3}^{\prime }\;and\;\prime BS_{3}\prime$ *i.e., ‘{* $BS_{2},BS_{3}$ *}’ is known as an itemset (or a pattern). This pattern contains two items. Therefore, it is a length-2 pattern. The pattern ‘{* $BS_{2},BS_{3}$ *}’ appears in the epochs having ids 3, 4, and 7. Therefore,* $cov_{BSD}\left(BS_{2},BS_{3}\right)$ *= {3, 4, 7}. Hence,* $Sup_{SDB}\;\left(BS_{2},BS_{3}\right)$ *= |{3,4,7}| = 3. If the user-defined* minSup(ε) *= 3, then ‘{* $BS_{2},BS_{3}\}$ *‘ is a frequent pattern as* $Sup_{SDB}\;\left(BS_{2},BS_{3}\right)$ *≥* minS *.*

**Definition 1** (a period of X)**.** Let $m_{j+1}^{X}$ and$\;m_{j}^{X}$; j ∈ [1, (m − 1)] be two consecutive timeslot-ids of pattern X in BSD. Then, $p_{j}^{X}=\;m_{j+1}^{X}-\;m_{j}^{X}$ (i.e., the number of timeslots) is defined as the j-th period of X in BSD. As mentioned in [25], a ‘null’ epoch with no sensor data is considered in the period computation at the beginning of BSD, i.e., $t_{p}$. = null, where $t_{p}$ is the first epoch in the pattern occurrence list. Similarly,$\;t_{n}$, is the n-th epoch in BSD, i.e., the last epoch to be considered.

**Example** **3.** Continuing with Example 2, the pattern ‘ $BS_{2},\;BS_{3}$’ has appeared in the TID of 3, 4, and 7. Therefore, a period for ‘ $BS_{2},BS_{3}$’ is 3 (= 3 − $t_{p}$), 1 (= 4 − 3), 3 (= 7 − 4), 0 (= $\;t_{n}-7$) where $t_{p}$ = 0, $\;t_{n}=7.$

**Definition 2** (The interested-recurrence period of pattern X)**.** Let$\;Per\left(X\right)=\left\{\;P_{1}^{X},\;P_{2}^{X},\ldots \ldots ,\;P_{r}^{X}\right\}$, where r is the total number of periods of X in BSD, be the complete set of all the periodic occurrences of X in TDB. A $P_{j}^{X}$∈Per(X) is said to be the periodic-recurrence iff $P_{j}^{X}\;less\;than\;or\;equal\;maxPer,$ where maxPer (ɛ) is the largest occurrence interval defined by use.

**Example** **4.** From Example 3, the complete set of periods for the pattern ‘$BS_{2},BS_{3}$’ is $Per\left(BS_{2},BS_{3}\right)=\left\{\;3,\;1,\;3,\;0\right\}$ if the user-defined maxPer(ε) = 2; then, $P_{2}^{BS_{2},BS_{3}}$ and $P_{4}^{BS_{2},BS_{3}}$ are the periodic-recurrence of the patterns but $P_{1}^{BS_{2},BS_{3}}$ and $P_{3}^{BS_{2},BS_{3}}$ are not periodic-recurrence as their value ≰ maxPer(ε).

In order to solve the problem of finding periodic patterns that may have acyclic repetition in the BSN database, we add a measure to check the number of interested periods in an itemset, which is:

**Definition 3** (The minimal-itemset occurrence)**.** Let $IP^{X}\subseteq \;Per\left(X\right)$ be the set of interested periods such that $\forall p\;\in \;IP^{X},p\;\leq \;maxPer\left(\varepsilon \right).$ The minimal-itemset occurrence, say MPR(x), is the size of $IP^{X}$, that is, MPR(x) = |$IP^{X}$ |.

Tanbeer [28] and all the extension work considered the periodicity of the pattern to be the maximum period in maxPr(X), that is, periodicity (X) = $max\;(P_{j}^{X}$|∀ $P_{j}^{X}$∈Per(X)). A drawback of maximum periodicity is that this measure may be too strict as the periodic pattern is discarded automatically if one of its periods is of a length greater than the maximum periodicity threshold defined by the user. To provide more flexibility in evaluating pattern periodicity and overcome this limitation of traditional PFP mining algorithms, the concept of the average interested-period is proposed, which is defined as follows.

**Definition 4** (Average interested-period of an itemset X)**.** *Let $IP^{X}\subseteq \;Per\left(X\right)$ be the set of interested periods such that$\forall p\;\in \;IP^{X},p\;\leq \;maxPer\left(\varepsilon \right).$ The periodicity of X is defined as $avgPer\left(X\right)=\sum_{p\;\in \;IP^{X}}/\left|IP^{X}\;\right|$.*

For example, let the interested period of itemset $\prime BS_{2},BS_{3\;}^{\prime }$be$\;IP^{BS_{2},BS_{3}}$ = {1, 0} and its average periodicity is $avgPer\left(BS_{2},BS_{3}\right)=0.5$

. Although the usefulness of considering average periodicity as a measure of the period length of an itemset ensures that we consider any partial or full periodic pattern without any restriction, we cannot directly evaluate the periodicity of a pattern with similar periods or even use the average as a sole measure because Definition 2 does not consider whether an itemset has an occurrence’s periods that are similar or vary widely. For instance, consider an itemset with the given $IP^{\mathrm{BSx}}$ = {1, 3, 5, 7} and $IP^{\mathrm{BSy}}$ = {10, 10, 10, 10, 10, 10, 10, 10}. Although itemsets BSx and BSy have totally distinct periods, avgPer(*x*) = avgPer(y) = 8. Hence, using only the average as a sole periodicity measure is misleading and will not solve the problem of reporting patterns with similar periods. The work in [29] solved this issue by combining the average periodicity measure with the standard deviation measures (s). Here, we use the same solution, but we restrict our periodicity only to the interested periods that match the user’s request.

**Definition 5** (Problem Definition)**.** *We have a BSN database BSD, user-defined minimum support threshold ε, maximum period maxPrd, minimal-itemset occurrence threshold MPR, periodicity measure per difference factor p1, pattern X, and interested period $IP^{X}$. X is a periodic-frequent pattern if $Sup_{SDB}$(X) ≥ ε, |$IP^{X}$| ≥ MPR, (per − p1) ≤ avgPer($IP^{X}$) − std($IP^{X}$) and avgPer($IP^{X}$) + std($IP^{X}$) ≤ (per + p1).*

With Definition 5, we report every periodic pattern that has either cyclic or acyclic occurrences with similar regular periods in the BSN database. Many diseases may have some set of values that occurs at the same time and later disappears and then reappears, for example. Supposing disease X results in increases in body temperature and in heart rate beats three times within one month, the patient may then face a further disease symptom. Moreover, how we can detect the relationships between different BSN reading parameters and ensure that the generated patterns are not due to random occurrences is a major issue in analyzing the vital signs obtained from BSNs. To enable reporting only the periodic-frequent physiological parameters that are vital for decision making, we test the positive correlations among them using the productive-associated test as proposed in [29] as follows.

**Definition** **6.** *A periodic frequent pattern, X in BSD, is a productive pattern if, for all X_1_, X_2_ such that, (X_1_ ⊂ X), (X_2_ ⊂ X), (X_1_∪X_2_ = X), and (X_1_∩X_2_ = ∅), then,*

(3) $$\left(\frac{\left|BSD\right|-avgPer\left(X\right)}{avgPer\left(X\right).\left|BSD\right|}\;\right)>\left(\frac{\left|BSD\right|-avgPer\left(X_{1}\right)}{avgPer\left(X_{1}\right).\left|BSD\right|}\;\times \;\frac{\left|BSD\right|-avgPer\left(X_{2}\right)}{avgPer\left(X_{2}\right).\left|BSD\right|}\right)$$

Our productive pattern test in Definition 6 is the same as the productivity test proposed in [29,49] as follow: For any periodic itemset$\;X_{n}$, $\frac{\left|BSD\right|-avgPer\left(X_{n}\right)}{avgPer\left(X_{n}\right).\left|BSD\right|}\;$ can be re-written as $\frac{\left|BSD\right|-avgPer\left(X_{n}\right)}{avgPer\left(X_{n}\right)}\;\times \frac{1}{\left|BSD\right|}$ where $\frac{\left|BSD\right|-avgPer\left(X_{n}\right)}{avgPer\left(X_{n}\right)}=\;\frac{cov_{BSD}\left(X_{n}\right)}{\left|BSD\right|}=Sup_{SDB}\left(X_{n}\right)$. Hence our productive-association test can be expressed as utilized in [49] as:

(4) $$\left(\frac{\left|BSD\right|-avgPer\left(X\right)}{avgPer\left(X\right).\left|BSD\right|}\;\right)>\left(\frac{\left|BSD\right|-avgPer\left(X_{1}\right)}{avgPer\left(X_{1}\right).\left|BSD\right|}\;\times \;\frac{\left|BSD\right|-avgPer\left(X_{2}\right)}{avgPer\left(X_{2}\right).\left|BSD\right|}\right)\;=Sup_{SDB}\left(X\right)>\;Sup_{SDB}\left(X_{1}\right)\times Sup_{SDB}\left(X_{2}\right)$$

Through Definition 6, a periodic-frequent pattern, X in BSD is productive if every subset itemset formed with an inherent item association in BSD ensures that all reported patterns are correlated and not due to random occurrences. The productive-associated test satisfies the downward closure property that every superset of a productive itemset will always contain a productive itemset, and hence we use it as one of our pruning strategies for eliminating non-productive itemsets from the reported periodic-frequent patterns.

## 5. Mining Productive-Associative Periodic-Frequent Patterns

Here, we propose an efficient PPFP-tree data structure with one database scan over the BSN database to mine the complete set of periodic patterns that has productive association.

### 5.1. PPFP-Tree Structure

The PPFP-tree has a root node referred to as the ‘null’ and a set of prefix trees. It also has a header table called the body sensor data table (BSD-table). The BSD-table consists of five fields (*i*, *f*, *r_c_*, *L_t_*, *p*): (i) body sensor name (*BSi*); (ii) frequency count of *BSi*; (iii) periodicity of *BSi*; (iv) last occurrence *tid* of *BSi*; and (v) a pointer to the first node in the PPFP-tree for each sensor value. After building the PPFP-tree and traversing it once, we calculate the periodicity (*r_c_*) and *L_t_* for each sensor. The prefix tree structure is similar to Han’s [21] prefix tree used to mine the Frequent Pattern-tree (FP-tree). However, the nodes in the PPFP-tree do not maintain the support count. Instead, they maintain the occurrence information of each sensor in the BSN database by keeping each sensor timeslot only at the last node of every epoch. The prefix tree has two types of nodes: ordinary nodes and tail nodes. The former is similar to the FP-tree ordinary node, whereas the latter is the node that represents the last item of any sorted epoch. The structure of the tail node is of the form Nj [t1, t2, ..., tn], where Nj ∈ X is the sensor’s node name and t_n_ ∈ TID is the timeslot-id of an epoch in which Nj is the last sensor. To ensure a high degree of compactness in the PPFP-tree, items are arranged in support-descending order. Both nodes maintain parent, children, and node traversal pointers to fasten the tree traversing process for sensor *BSi*.

### 5.2. PPFP-Rree Construction

The construction of *a PPFP-tree* is similar to the frequent pattern tree and regular pattern tree [21,26]. However, we use a single database scan over the sensor data, and captures the complete sensor database information in a compact manner.

**Algorithm 1:** PPFP-Tree construction. 
**Input**: The sensor database BSD;
**Output**: An PPFP-tree;		  
    1:   **Begin**		  
    2:     Create the root R of an PPFP-tree, Tree, and label it ‘null’.
    3:   	 for each epoch ti ∈ BSD do
    4:            **if** ti ≠ NULL **then**
    5:               for each item tcur ∈ ti  do
    6:                    **if** tcur.f = 0 **then**          /*  it’s first occurrence */
    7:                      Set tcur.f = 1 and lt = tcur
    8:                    else
    9:                      **if** tcur- lt <= maxPrd **then**
    10:                         add tcur- lt to tcur.pr
    11:                    **end if**
    12:                Set ++f and lt = tcur.
    13:              **end if**
    14:                Add candidate items of ti to BSD-list.
    15:               **end if**
    16:           **end for**
    17:               Select and sort the candidate items in BSD-list in support descending order.
    18:                Call Insert_PPFP_tree(BSD-list, Tree).
    19:          **end for**
    20:    Update BSD- table.
    21:    Call PPFP-growth (PPFP-Tree, null);
    22:   **End**
	


The PPFP-tree construction process initialized with a root node ‘null’. Using the BSD-list, we perform a single scan on the database to discover single items and their support inside the sensor database. Afterward, the BSD-table is created. The BSD-table is in descending order to ensure tree compactness. Moreover, only the items that satisfy the minimum user support threshold will take part in the construction of the PPFP-tree. Let *tcur* represent the sensor timeslot of the current epoch. A temporary array *lt* explicitly records the last occurring epochs of all patterns in the BSD-list. Let us visit a construction example for the database given in Table 3 by following Algorithm 1.

A root node with ‘null’ (line: 2) is the first step in the tree building process. Next, we scan the database once and get the support of each single item (lines: 3–14); then, we sort the items listed in support-descending order. The construction of the tree starts with a call to the procedure on line 19 by executing the *Insert_PPFP_tree* procedure given in Algorithm 2.

**Figure 2:**
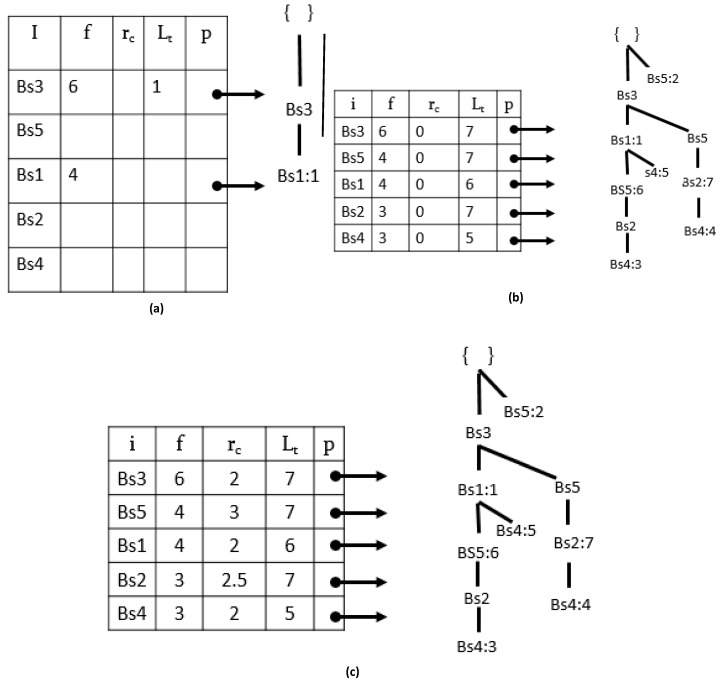
PPFP-tree construction with *MinSup* = 3, *Maxpe r* = 3, *MPRD* = 2. (**a**) PPFP-tree after inserting TID = 1, (**b**) PPFP-tree after inserting all BSD epochs, (**c**) Final PPFP-tree.

The tree construction starts by adding the first epoch {1: Bs3, Bs1}, according to the BSD-list order, as shown in Figure 2a. The *tid* occurrence value of the epoch is saved in the tail node ‘*Bs1*:1’ (line 11). The process is repeated recursively for the other epochs in the database. Figure 2b shows the PPFP-tree constructed after scanning the second epoch, third epoch, and entire database. For the simplification of the figures, the node traversal pointers are not shown. 

**Algorithm 2:** Insert_PPFP_tree (BSD-list, Tree). 
    1:   Let [l|L], where l is the first sorted epoch and L is there remaining epoch in the given list.
    2:   while l is non-empty  do
    3:      **if** l has child N such that l.sensorName ≠ N.sensorName **then**
    4:          Create a new node N.
    5:        Let its parent node be linked to Tree.
    6:        Let its node-link be linked to nodes with the same item Name via the node-link structure.
    7:               **if** l is the tail-sensor of BSD-list **then**
    8:                    **if** N = an ordinary node **then**
    9:                       assign a tid-list to N;
    10:          **end**
    11:        add the tid of BSD-list in N's tid-list;                    
    12:       **end if**
    13:    **end while**
    14:   Remove l from L.
    15:   call Insert_PPFP-tree(L, N);
              


Once the PPFP-tree is constructed, we use the pointers of each sensor from the BSD-table in order to scan the tree and calculate the values given in Definitions 3 and 4 for each sensor in the BSD-table (line: 20). 

To efficiently complete this process, all *tid*(s) at each sensor tail node are accumulated in a temporary array for each sensor in the BSD-table by traversing the whole tree once. During the *tid* accumulation process, we begin in BSD-table in reverse order, i.e., we start from the last sensor of the BSD-table.

Continuing with our running example, Figure 2c shows the final PPFP-tree status and the BSD-table with the periodicity (*rc*) and last *tid* (*Lt*) of each sensor. Therefore, with a single BSD scan, the PPFP-tree maintains all BSD information in a compact manner. 

Once the PPFP-tree is constructed, an efficient FP-growth-inspired pattern mining technique is employed to find the complete set of productive-associated periodic-frequent patterns from the current database (line: 21). In the next subsection, we discuss the productive-associated periodic-frequent pattern mining process from the PPFP-tree.

### 5.3. Mining the PPFP-Tree

We mine the PPFP-tree recursively in decreasing size using a pattern-growth approach by creating conditional pattern-bases and corresponding conditional trees without constructing any additional database scans. While constructing the prefix tree, we map the *tid* list of every node of ‘Si’ to all its path items.

Algorithm 3 shows the construction of the prefix-tree in PPFP-growth. After we construct the tree and all of its nodes, we have to execute the call to Algorithm 3. Then, we choose the last sensor ‘*Bsi*’ from the BS-table (line 1). After that, we construct its prefix-tree (line 2).

**Algorithm 3:** PPFP-growth (tree, α). 
    1:   **for** each sensor S_α_ in the header of Tree in reverse-order do
    2:       Generate pattern S_β_ = *X_i_* ∪ S_α_. map all *X_i’_s* t_id_-list to temporary arrays (*TS*β) for all sensors in *Sα***.**
    3:       **if** *TS*β.*Size* ≥ *minSup(ε)* and S_β_  Productive according to Definition 6 **then**
    4:          Call *CalculateInterestedPeriodicity* (*TS*β).
    5:           **if**  S_β_  is periodic according Definition 5 **then**
    6:              Construct first S_β_ conditional pattern base then S_β__Tree conditional PPFP-tree.
    7:                     **if** *Tree* S_β__Tree = ∅ **then**
    8:                             Call PPFP-growth(S_β__Tree, S_β_);
    9:                     **end if**
    10:            **end if**
    11:       Delete the node *X_i_* from the *Tree* and push the *X_i_*’s t_id_-list to its parent nodes.
    12:    **end for**



Then, we check the productivity of the new itemset and check its support value (line 3). If the pattern is productive with Definition 6, we then call Algorithm 4 to check the pattern periodicity.

**Figure 3:**
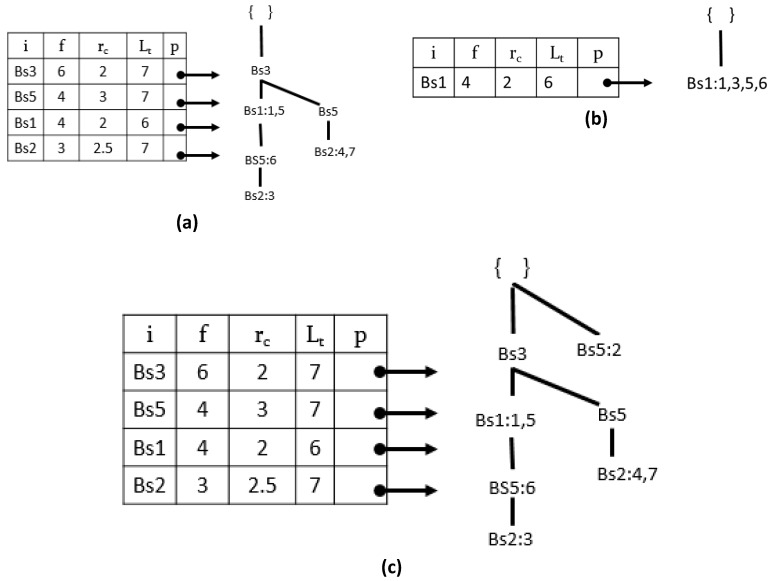
Prefix-tree and conditional tree construction with the PPFP-tree. (**a**) Prefix-tree for ‘*Bs4*’ (**b**) Conditional tree for ‘*Bs4*’ and (**c**) PPFP-tree after removing item ‘*Bs4’*.

Figure 3a shows the prefix-tree of ‘Bs4’. Figure 3b shows the conditional tree of ‘Bs4,’ the status of the PPFP-tree after removing the last item ‘Bs4’ from the BSD-table shown in Figure 3c, assuming that the user defines *maxper* = 3 *avg* = 1.4 *diff* = 0.8.

In Algorithm 4, the *tid* list of the construct node is used to calculate the set of an interesting pattern period. The period is considered if its values are no greater than the user-given minimum periodicity threshold (line 3). The pattern’s average periodicity and standard deviation are calculated. Continuing with the ‘Bs4’ pattern, we find that the pattern ‘Bs4, Bs1’ is productive. A recursive process of creating the prefix-tree and its conditional tree is repeated for further extensions of ‘ij’ until BS-table = ∅.

**Algorithm 4:** CalculateInterestedPeriodicity (*TSβ*: an array of timeslot-ids containing S).
    1:   **Begin**
    2:   Set IP = −1 minpr = 0 and IPcur = *TSβ*[0].               /* subtract 0 from the first value (*TSβ*[0] − 0).*/
    3:       **if** IPcur > maxPer **then**
    4:          add IPcur to IP.                      /* list of interested period*/
    5:      **end if**
    6:   **for** i = 1; i < *TSβ.size* − 1;++i do
    7:       Calculate IPcur = *TSβ*[*i* + 1] − *TSβ[i]*.
    8:           **if** IPcur > maxPer then
    9:             add IPcur to IP.
    10:           **end if**
    11:   **end for**
    12:   Calculate IPcur = |SDB| − *TSβ* [*TSβ.size*], and repeat the steps numbered from 8 to 10.
    13:    **If** *IP*.*size* ≥ *MPRD* **then**
    14:    Calculate average and Standard Deviation of IP.
    15:   **end**



## 6. Experimental Study

- The experiment was carried out on a 64-bit Core i5 processor running Windows 10, and with 12 GB of free RAM. We selected three benchmark datasets, including both synthetic and real-world databases. The datasets were chosen because they represent the main characteristics of the vital parameters and sensors’ data (dense, sparse, and long transactions), as shown in Table 4. In our experimental analysis, we implement the following algorithms:
- PPFP-growth is our implementation of the mining algorithm based on Definitions 5 and 6. PPFP reports the productive-associated full/partial periodic-frequent pattern using a pattern-growth mining approach with one database scan, and we use the productive measure to report only the periodic-frequent patterns with pattern associations. At the same time, this accelerates the process of mining.
- CPFP is our implementation of the work presented in [28]. CPFP finds the periodic-frequent pattern that occurs in acyclic or cyclic database repetition without the productive measure and within two database scans.
- PPFP is our implementation of the work in [29] that reports productive periodic-frequent patterns using an a priori-like approach [40].

Synthetic databases are used frequently to evaluate frequent-pattern mining algorithms. Those datasets generated by using the IBM data generator [40] and the real dataset are used from SPMF [50,51,52]. All mentioned algorithms are implemented in Java. 

The experiments consisted of two parts. First, we compare the PPFP-growth algorithm with the CPFP algorithm. Second, we compare the PPFP-growth algorithm with PPFP.

### 6.1. Comparing the Execution Time of the PPFP-Growth Algorithm and CPFP Algorithm

In the first part of the experiments, the PPFP-growth algorithm and CPFP algorithm were run on each dataset with fixed *MPR*, periodicity, and range values, while varying the *minSup* and *maxPer* parameters. To be able to compare PPFP-growth with CPFP, CPFP was run with the value calculated by PPFP-growth. Execution times were measured for each algorithm using the Java API.

For each dataset, the values for *MPR*, *periodicity*, and *range* are dataset-specific (i.e., they were found empirically for each dataset) and are chosen to show the trade-off between the execution time of each algorithm. Additionally, the notation PPFP-growth X-W-Y represents the PPFP-growth algorithm with *MPR* = X, *periodicity* = W, and *range* = Y. 

**Figure 4:**
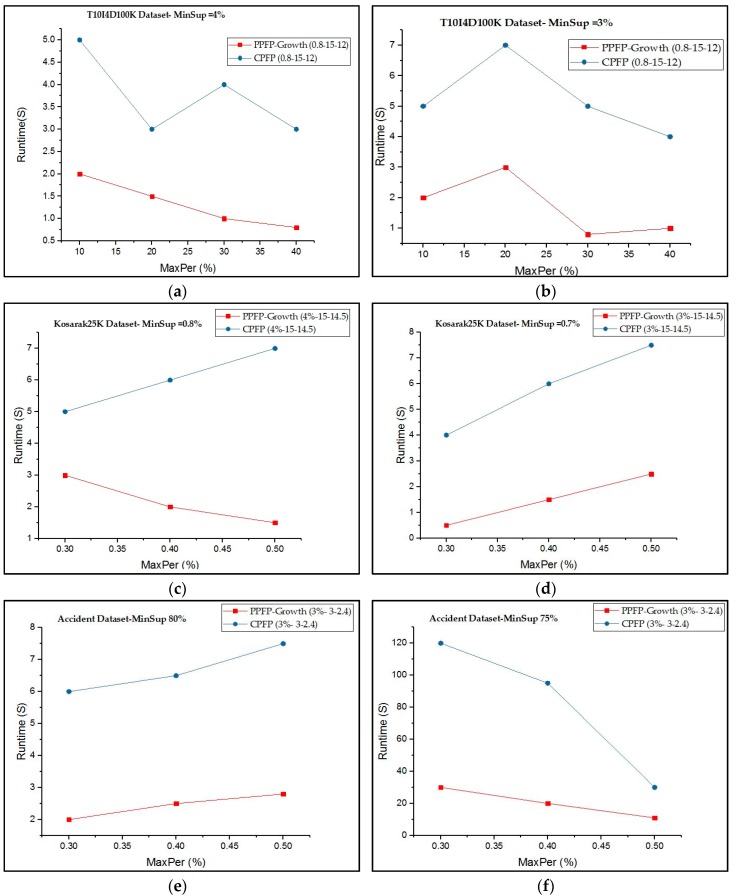
Execution times of PPFP-growth and CPFP. (**a**) Execution time on *T10I4D100K* with *MinSup* = 4%. (**b**) Execution time on *T10I4D100K* with *MinSup* = 3%. (**c**) Execution time on *Kosarak25K* with *MinSup* = 0.8%. (**d**) Execution time on *Kosarak25K* with *MinSup* = 0.7%. (**e**) Execution time on *accident* with *MinSup* = 80%. (**f**) Execution time on *accident* with *MinSup* = 75%.

Figure 4 compares the execution time of our proposed algorithm with that of the CPFP for mining all periodic-frequent patterns that occur in all or part of the database with respect to the user-given X-W-Y values. It can be observed that mining PPFP-growth is generally much faster than mining periodic items using the CPFP algorithm. 

Considering all the datasets, PPFP-growth is up to four times faster than CPFP depending on the parameters. The reason is that the search space is huge when we try to find all periodic patterns without using the productivity test. Moreover, CPFP uses two database scans. Additionally, the PPFP-growth algorithm only searches for productive-associated periodic patterns and thus prunes a large part of the search space containing non-productive periodic patterns. For the *accident* dataset, PPFP-growth can be up to four times faster than CPFP depending on the parameters. However, it starts to have the same runtime, in some cases. The reason is that the dataset is sparse and the cost of calculating productivity can compensate for the cost of pruning the search space.

### 6.2. Comparing the Execution Time of the PPFP-Growth Algorithm and PPFP Algorithm

In the second part of the experiments, to compare the execution time of the PPFP-growth algorithm and the PPFP algorithm, we fixed the *minSup*, *maxPer*, and *MPR* parameters for the ‘T10I4D100K’ and ‘accident’ datasets, while varying the *periodicity*(*per*) and *range* (*diff*) values. However, for the ‘Kosarak25K’ dataset, we changed the *minSup* values and fixed the *maxPer*, *MPR*, *per*, and *diff* values. Furthermore, the parameter values are dataset-specific. Additionally, the notation PPFP-growth X-W-Y for the *Kosarak25K* dataset represents the PPFP-growth algorithm with *MPR* = X, *periodicity* = W, and *range* = Y. We also used the calculated value (*periodicity*, *diff*) of PPFP-growth to show the execution time of the PPFP algorithm.

**Figure 5:**
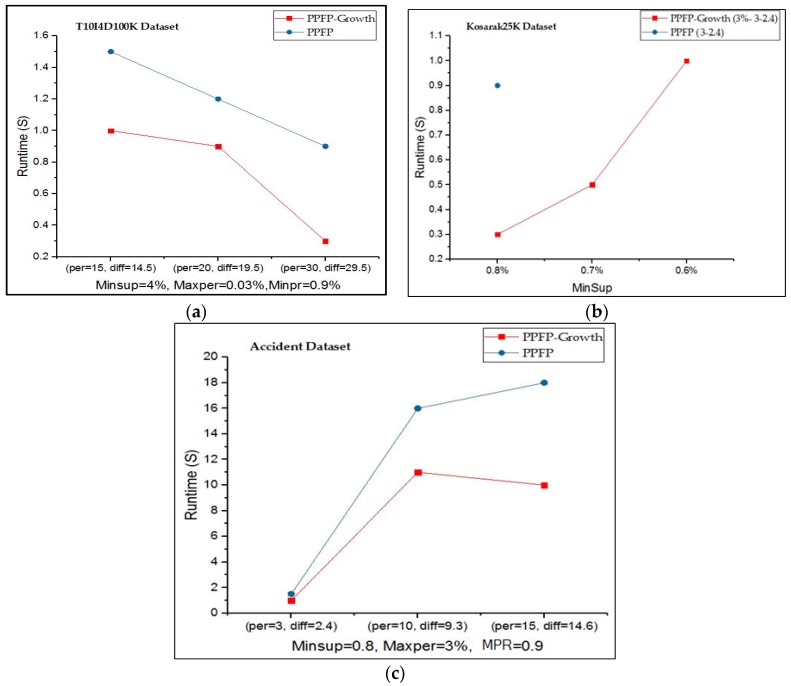
Execution time of PPFP-growth and PPFP. (**a**) Execution time on *T10I4D100K* dataset. (**b**) Execution time on *Kosarak25K* dataset. (**c**) Execution time on *accident* dataset.

In Figure 5a–c, we compare the execution time for the algorithms of PPFP-growth and PPFP. It can be observed that using the PPFP-tree data structure with a single database scan in the PPFP-growth algorithm outperforms the Apriori-based PPFP execution time depending on the parameter values. For the *Kosarak25K* dataset, no results are shown for PPFP because it cannot terminate within 1000 s, while PPFP-growth terminates in less than seconds. The reason is that when decreasing *minSup* in these datasets, the search space increases, which increases the delay for discovering the patterns depending on the generate-and-test PPFP algorithm. On the other hand, PPFP-growth still terminates on these datasets because the PPFP-tree avoids the combinatorial explosion problem of candidate generation as in a priori-like algorithms [40].

## 7. Conclusions and Future Work

Demand for new technologies to promote and assist significant decision making in a smart health system has been greatly boosted in the past few years. Here, we provide a new pattern-mining algorithm to mine the productive-associated PFPs from health-related information collected from smart homes to promote important decision making in healthcare. We present the interested pattern measures to identify the interesting PFP set of periods and a measure to identify the set of productive-associated PFPs. We also develop PPFP-growth, an efficient algorithm for mining the set of productive-associated PFPs. In the future, we plan to develop a context-aware abnormal human behavior algorithm in which the patient’s vital signs are analyzed with respect to other human activity data such as sitting, sleeping, or exercising. Context-aware multiple stream data from different home sensors could reveal more accurate patterns regarding human health, as vital signs are sometimes affected by human activity. The heart rate could increase when exercising if we have not considered this context situation, meaning a false alarm could be generated. In addition, we would like to investigate alternative techniques to further reduce the computational cost of mining productive patterns.

## Figures and Tables

**Table 1:** The five vital signs and their sensor acronyms used in this example.

Bio-Signal	Sensor Acronym	Range (beats/min)			
Heart Rate	HR	Very High	High	Normal	Low
		above 100	70–99	40–69	below 40
Respiratory Rate	RR	**Range (breaths/min)**			
		Very High	High	Normal	Low
		21–25	15–21	12–15	Below 5
Blood O_2_ Saturation	SO_2_	**Range Percentage (%)**			
		High		Normal	Low
		95–100		80–90	Below 83
Diastolic Blood Pressure	DBP	**Range (mmHg)**			
		Very High	High	Normal	Low
		above 110	90–109	65–84	35–59
Body Temperature	BT	**Range (°C)**			
		Very High	High	Normal	Low
		above 40	39–39.9	37–38	36–36.9

**Table 2:** Comparison of the issues addressed by our approach against current related work. ‘Issue 1‘ discovers full or partial patterns, ’Issue 2‘ is the ability to do this based on one database scan, and ‘Issue 3‘ represents the ability to generate correlated periodic-frequent patterns. The symbol ‘✔’ indicates that the issue addressed and ‘✖’ indicates that the issue is not addressed by the corresponding work.

	Issue 1	Issue 2	Issue 3
[21]	✖	✖	✖
[26]	✖	✖	✖
[25]	✖	✔	✖
[28]	✔	✖	✖
[48]	✔	✖	✖
[29]	✖	✖	✔
Our approach	✔	✔	✔

**Table 3:** A Sensor Database (SDB).

Id	Epoch	Id	Epoch	Id	Epoch
1	$BS_{1},\;BS_{3}$	4	$BS_{2},BS_{3},BS_{4},BS_{5}$	7	$BS_{2},BS_{3},BS_{5}$
2	$BS_{5}$	5	$BS_{1},BS_{3},BS_{4}$		
3	$BS_{1},BS_{2},BS_{3},BS_{4},BS_{5}$	6	$BS_{1},BS_{3},BS_{5}$		

**Table 4:** Dataset characteristics.

Dataset	Type	Transactions Number
T10I4D100K	Synthetic	100,000
Accident	Real sparse, many items	7593
Kosarak25K	Real dense, long	25,000
